# Characterization and Full Genome Sequence of Novel KPP-5 Lytic Phage against *Klebsiella pneumoniae* Responsible for Recalcitrant Infection

**DOI:** 10.3390/biomedicines9040342

**Published:** 2021-03-28

**Authors:** Ahmed R. Sofy, Noha K. El-Dougdoug, Ehab E. Refaey, Rehab A. Dawoud, Ahmed A. Hmed

**Affiliations:** 1 Botany and Microbiology Department, Faculty of Science, Al-Azhar University, Nasr City, Cairo 11884, Egypt; ehabrefaey@azhar.edu.eg (E.E.R.); ahmed_hmed@azhar.edu.eg (A.A.H.); 2 Botany and Microbiology Department, Faculty of Science, Benha University, Benha 13518, Egypt; nohaeldougdoug@gmail.com; 3 Virus and Phytoplasma Research Department, Plant Pathology Research Institute, Agricultural Research Center (ARC), Giza 12619, Egypt; rdawood@jazanu.edu.sa; 4 Department of Biology, Faculty of Science, Jazan University, Box 114, Jazan 45142, Saudi Arabia

**Keywords:** Bacteriophage, *Autographviridae*, *Klebsiella pneumoniae*, Multidrug resistance, phage therapy, Full genome, Bioinformatics

## Abstract

*Klebsiella pneumoniae* is a hazardous opportunistic pathogen that is involved in many serious human diseases and is considered to be an important foodborne pathogen found in many food types. Multidrug resistance (MDR) *K. pneumoniae* strains have recently spread and increased, making bacteriophage therapy an effective alternative to multiple drug-resistant pathogens. As a consequence, this research was conducted to describe the genome and basic biological characteristics of a novel phage capable of lysing MDR *K. pneumoniae* isolated from food samples in Egypt. The host range revealed that KPP-5 phage had potent lytic activity and was able to infect all selected MDR *K. pneumoniae* strains from different sources. Electron microscopy images showed that KPP-5 lytic phage was a podovirus morphology. The one-step growth curve exhibited that KPP-5 phage had a relatively short latent period of 25 min, and the burst size was about 236 PFU/infected cells. In addition, KPP-5 phage showed high stability at different temperatures and pH levels. KPP-5 phage has a linear dsDNA genome with a length of 38,245 bp with a GC content of 50.8% and 40 predicted open reading frames (ORFs). Comparative genomics and phylogenetic analyses showed that KPP-5 is most closely associated with the *Teetrevirus* genus in the *Autographviridae* family. No tRNA genes have been identified in the KPP-5 phage genome. In addition, phage-borne virulence genes or drug resistance genes were not present, suggesting that KPP-5 could be used safely as a phage biocontrol agent.

## 1. Introduction

*Klebsiella pneumoniae*, a Gram-negative bacterium, a facultative anaerobic, is the primary cause of serious infections in humans around the world [1]. It is well-known that many nosocomial infections are caused by it, leading to increased morbidity and mortality rates [2,3]. In addition, *K. pneumoniae* has been considered to be an important food-borne pathogen found in many food types [4,5,6]. Unfortunately, *K. pneumoniae* exhibits a high resistance to many antimicrobials due to the presence of resistance genes, and many other resistances are encoded in plasmids that make treatment difficult [7].

In many habitats, including the human body, bacteria can form biofilms to face the adverse impacts of environmental challenges [8]. Biofilms are known as surface-attached aggregates embedded in an extracellular polymeric substance matrix, consisting of polysaccharides, proteins, enzymes, DNA, lipids, and water [8,9]. The ability of *K. pneumoniae* to form a biofilm is confirmed by numerous evidences, and a lot of information has supported that such behavior plays a key role in the acquisition of antibiotic resistance [10].

In the current scenario of emerging antibiotic resistance, the search for alternatives to antibiotics is increasing, especially bacteriophage therapy, which has gained considerable importance [9,11,12,13,14]. Phages appear to be a promising tool to lyse pathogenic bacteria, causing their destruction [15]. In addition, phages have been shown to be active not only against plank-tonic bacteria but also against bacteria organized in biofilms by lysing bacterial cells or the biofilm matrix [16,17]. Phage proteins, such as endolysins, hydrolases, depolymerases, and holins, are already extensively investigated in the development of promising new antibiotics, where phages use these enzymes to infect or lyse bacteria [18,19]. Even more phage proteins are directly linked to bacterial proteins in order to sustain a successful infection cycle, and, surprisingly, the functional mechanisms of these associations are largely unknown [18,20].

Here, we report and describe the genome and basic biological characteristics of a novel phage capable of lysing *K. pneumoniae* that is resistant to many antibiotics.

## 2. Materials and Methods

### 2.1. Bacterial Strain for Bacteriophage Isolation

A food isolate of *K. pneumoniae* strain CFS17 previously isolated from a white cheese sample (Plant Viruses and Bacteriophage Lab of Botany and Microbiology Department, Faculty of Science, Al-Azhar University, Cairo, Egypt) was used as a host for isolation and characterization of bacteriophage. Identification of the host isolate was also confirmed using the automated Biomerieux VITEK 2 system according to Funke and Funke-Kissling [21,22]. Antibiotic resistance of this strain has been shown to be resistant to many antibiotics used commercially in both human and veterinary medicine. In this test, 17 antibiotic disks (Oxoid, UK) of different groups of antibiotics were investigated for their potential effect against the host isolate. The targeted antibiotics and their patterns are included in Table S1. The test was done according to standard Kirby-Bauer disk diffusion method [23], and the interpretation of the data was performed by National Committee for Clinical Laboratory Standards “NCCLS” standardized protocols and designated as R (resistant), I (intermediate sensitive), and S (sensitive) [24].

### 2.2. Inoculum Preparation of the Host Isolate

The bacterial host strain was cultured in Tryptic Soy Broth (TSB, Difco^TM^, BD, USA) media and incubated at 37 °C to the mid-logarithmic phase. The concentration of cells was adjusted using CFU/mL compared to the 0.5 McFarland turbidity standard (1.5 × 10^8^ CFU/mL) [25].

### 2.3. Bacteriophage Isolation and Purification

The *K. pneumoniae* phage was isolated from sewage water collected from the Wastewater Treatment Plant (Kafr El-Sheikh Governorate, Egypt) using the Bibi et al. [26] method. Sewage sample was centrifuged; 10 mL of the supernatant sample was added to the 10-mL TSB media (Difco^TM^, BD, USA). One hundred microliters of the mid-logarithmic phase fresh culture of the host bacteria was inoculated and then incubated at 37 °C for 24 h in the shaker incubator (270 rpm). After incubation, the suspension was centrifuged and the supernatant filtered through a 0.45-μm filter and stored at 4 °C. The previous filtrate was spotted on Trypticase Soy Agar (TSA) plates in order to detect the bacteriophages, and the agar overlay method was performed for phage isolation, purification, and titration in accordance with Sangha et al. [27]. Ten-fold serial phage lysate dilutions (1–10^−9^) were performed in TSB; each dilution was mixed with the host culture and placed at 37 °C in the incubator for 15 min. The incubated mixture was added to the 4-mL soft agar (0.7% agar) medium and poured onto the surface of the TSA plates, then incubated at 37 °C for 24 h, and the morphologies and sizes of the plaques were determined. For phage purification, the replating of captured plaques was frequently performed, and all dilutions for the phage titer determination were performed. The experiment was conducted in triplicate.

### 2.4. Host Range Determination

Host spectrum of isolated bacteriophages was determined by the spot-test assay according to Huang et al. [28] and Jun et al. [29] on 31 bacterial strains from different sources. The bacteriolytic activity was assayed against 19 strains of *Klebsiella pneumoniae* (clinical and food isolates), six of *Escherichia coli* (clinical isolates), three of *Salmonella* Typhimurium (food isolates), and 3 of *Pseudomonas aeruginosa* (clinical and food isolates). Three milliliters of soft agar (0.7% agar) medium was inoculated with 100 μL of test strain and poured onto Trypticase Soy Agar (TSA, Difco^TM^, BD, Franklin Lakes, NJ, USA) plates. The plates were left to solidify, and 10 μL of phage lysate was dropped on the surface of the previous plates and incubated at 37 °C for 24 h. After the incubation period, areas of clear spots were observed on the bacterial lawn. The host range of the most bacteriolytic phage (broadest host range) was also confirmed by the efficiency of plating method (EOP) in accordance with Mirzaei and Nilsson [30].

### 2.5. Transmission Electron Microscopy (TEM)

Stock KPP-5 phage was centrifuged at 16,000× *g* for 60 min and 4 °C after washing in a CM buffer. Supernatant was discarded, and the pellet was gently suspended in 20 μL of CM buffer; then, 5 mL was taken and placed on the carbon grids (200 mesh) coated with formvar and left for 2 min. The phage was stained by 2% uranyl acetate (negative stain), and the excess of the dye was removed by filter paper [31]. Sample examination in accordance with Accolas and Spillmann [32] was done at the Regional Center of Mycology and Biotechnology, Al-Azhar University, Cairo, Egypt using electron microscopy (Model Beckman 1010, Operated at 80 KV).

### 2.6. Determination of KPP-5 Phage Bacterial Culture Clearance Tendency

The potential effect of KPP-5 phage on the reduction of bacterial growth in the broth medium was evaluated using the Khawaja et al. [33] method. A host culture was incubated for 24 h at 37 °C; then, the culture (1 × 10^8^ CFU/mL) was added to 2 sterile clean flasks (100 mL) after incubation. KPP-5 phage (1 × 10^8^ PFU/mL) was added to one flask (test flask), while the second flask remained the same (positive control flask). In addition to the presence of a third flask containing only TSB media (negative control flask), it is indicated during the observations of the experiment. Flasks were incubated overnight in shaking (220 rpm) at 37 °C, while optical density (OD_620_) was recorded during the incubation period at 2, 4, 6, 8, 10, 12, and 24 h. The experiment was conducted in triplicate.

### 2.7. One-Step Growth Curve

The KPP-5 phage one-step growth curve against the host was carried out in accordance with Wang et al. [34]. KPP-5 phage (1 × 10^8^ PFU/mL) was added to a host fresh culture (1 × 10^6^ CFU/mL) at a multiplicity of infection (MOI) of 100. The mixture was placed in the incubator at 37 °C for 3 min, then centrifuged at 12,000× *g* for one minute to remove the free phages. The pellet was resuspended (time zero) in 100 mL of TSB medium and incubated at 37 °C. For 3 h, 5-min interval samples were taken, centrifuged, and the KPP-5 phage titer was calculated using the agar overlay method [27]. Burst size was detected by determining the ratio of the mean of virions released after bacterial infection to the mean of virions used at the beginning of the host infection. The experiment was conducted in triplicate.

### 2.8. Assessment of KPP-5 phage Thermal and pH Stability

The known KPP-5 phage titer (7 log_10_) was subjected to different temperatures (4 °C, 25 °C, 37 °C, 50 °C, 60 °C, and 75 °C) to determine its thermostability according to Philipson et al. [35]. Similarly, the same KPP-5 phage was incubated at various pH values (4, 5, 7, 9, and 11) in order to know its stability, as previously reported [36]. For the stability assessment, the KPP-5 phage titer was determined at 1, 2, 4, 8, 12, and 24 h using the above-mentioned agar overlay method [27].

### 2.9. Phage DNA Isolation and Genome Sequencing of KPP-5 Phage

Highly concentrated purified lysate was treated with RNase and DNase I Sigma Aldrich to digest any RNA or exogenous DNA. Then phage lysate was concentrated, and DNA was extracted using the Gene JET genomic DNA purification kit (Cat No. K0721, Thermo Scientific) according to the manufacturer’s instructions. Phage was whole-genome fragmented using restriction enzymes from the Ion Xpress plus library preparation kit (Thermo Fisher, USA); then, fragments were Nick repaired and adaptors ligated, and prepared libraries were barcoded with Ion Xpress Barcode Adaptors. After barcoding, a 200-bp library size was selected and purified using Agencourt beads (Beckman Coulter, Brea, CA, USA), and libraries were quantified using the Ion Library TaqMan Quantitation Kit (Cat No. 4468802, Life Technologies). The quantified libraries were pooled on molar equivalent rations to yield at least an average coverage depth of 150x–200x for each sample. The pooled libraries were clonally amplified using the Ion PGM Hi-Q view OT2 kit (Cat No. A29900, Life Technologies) according to the manufacturer′s instructions. Then, template ion sphere particles (ISP) were enriched using the Ion OneTouch ES system (Life technologies, USA). The positive ISP Quality was assayed on a Qubit 3.0 Fluorometer (Life technologies, Carlsbad, CA, USA) and then proceeded to perform the sequencing process. Using the Ion PGM Hi-Q View Sequencing Kit (Cat No. A30044, Life Technologies), all barcoded enriched samples were sequenced on the Ion Torrent PGM Platform (Ion Torrent PGM, Life Technologies, Carlsbad, CA, USA) on the Ion 316 Chip Kit V2 BC (Cat No. 488150, Life Technologies). The annotated complete genome sequence of KPP-5 phage was deposited in GenBank under accession number MW600722.

### 2.10. Bioinformatic Analysis of KPP-5 Phage Genome

Sequence reads were de novo assembled using trial Geneious prime software (version 11.0.6) using Geneious assembler with the default settings. The resulting scaffolds were filtered based on the length of the scaffolds. The whole assembly processes were repeated using unicycler workflow on galaxy (Galaxy Version 0.4.6.0) to ensure the reproducibility of the results with another assembler (SPAdes assembler 3.13.0). Prediction of genes was achieved by predicting open reading frames (ORFs) using ATG as starting codons with a minimum nucleotide length of 300 bp using the ORF finder in the National Center for Biotechnology Information (NCBI) database. The ORFs were translated and analyzed by SmartBlast and BLASTP to describe the potential functions and were further confirmed using the Pfam database [37]. SnapGene software was used to create a genome map of KPP-5 phage [38]. All ORFs were checked for genes encoding putative tRNAs using tRNA scan-SE version 2.0 (https://lowelab.ucsc.edu/tRNAscan-SE/) [39] and checked for genes encoding the potential protein-containing transmembrane domain using the TMHMM server v. 2.0 (https://www.cbs.dtu.dk) [40] with the default settings. ORFs were also analyzed using the databases of Virulence Searcher (http://www.mgc.ac.cn/VFs/) [41], ResFinder (https://cge.cbs.dtu.dk/services/ResFinder/) [42], and Antibiotic Resistance Genes (https://card.mcmaster.ca/) [43] to examine the virulence factor and drug resistance gene of the KPP-5 phage. The full-genome sequence of the KPP-5 phage was compared to the phage genome sequences in GenBank using BLASTN (somewhat similar sequences) in the NCBI database, and the most closely related phages were identified. The average nucleotide identity (ANI) values were calculated using the ANI calculator (http://enve-omics.ce.gatech.edu/ani/). The Circoletto program (http://tools.bat.infspire.org/circoletto/) [44] was used to visualize the phage comparative genome. CoreGenes 3.5 (http://binf.gmu.edu:8080/CoreGenes3.5/custdata.html) was used for the analysis of the phage-core genes where genes with a score greater than 75 were assumed to be the core genes [45]. The full-genome phylogenetic tree was created using the genome-BLAST distance phylogeny method in the Virus Classification and Tree Building Online Resource (VICTOR) [46]. In order to determine the taxonomic position of KPP-5 and to investigate its evolutionary history, a phylogenetic tree was constructed on the basis of conserved proteins frequently used in the analysis of the diversity, such as DNA polymerase, major capsid protein, and terminase large subunit. The clustalW option and the neighbor-joining method with 1000 bootstrap replicates implemented in Molecular Evolutionary Genetic Analysis (MEGA) software version 7.0 were used for multiple sequences alignment and phylogenetic tree construction, respectively.

## 3. Results and Discussion

### 3.1. Broad Host Range Bacteriophage Isolation and Morphology

**Figure 1:**
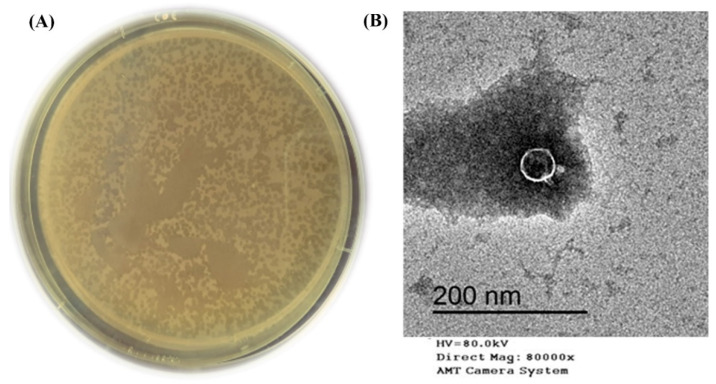
Plaques morphology (clear dots look like, small plaques, 1–1.5 mm diameter) formed by KPP-5 phage using overlay agar plate with *Klebsiella pneumoniae* lawn (**A**). Transmission electron micrograph image of *K*. *pneumoniae* KPP-5 phage podovirus morphology (**B**).

Six bacteriophages against multidrug resistance (MDR) *Klebsiella pneumoniae* were isolated from sewage water samples of the Wastewater Treatment Plant (Kafr El-Sheikh Governorate, Egypt). Only one bacteriophage isolate named KPP-5 showed clear plaques small in size (1–1.5 mm in diameter) that looked like dots (Figure 1A). The KPP-5 bacteriophage was purified, propagated, and titrated for further analysis. KPP-5 phage showed potent lytic activity, being able to infect all *K. pneumoniae* strains of different sources, with a high efficiency of plating (EOP) activity on 14 out of 19 strains (Table 1). On the other hand, KPP-5 could not infect any of the other *Escherichia coli*, *Salmonella* Typhimurium, and *Pseudomonas aeruginosa* strains tested (Table 1). *Klebsiella pneumoniae* is a dangerous opportunistic pathogen that is involved in many serious human diseases [47,48]. In addition, *K. pneumoniae* has been considered to be an important food-borne pathogen found in many food types [4,5,6]. Recently, MDR *K. pneumoniae* strains have recently spread and increased so that they have emerged as a cause of recalcitrant infections worldwide [49,50,51]. Bacteriophage therapy is therefore an effective alternative to multiple drug-resistant pathogens [52]. KPP-5 phage showed clear plaques, and this can be attributed to the production of soluble, polysaccharide-degrading enzymes [53]. Interestingly, the KPP-5 phage showed a broad host range where it could infect all the *K. pneumoniae* strains tested, although there was no lytic effect against the unrelated genes. On the contrary, the KP1513 and KP-34 *K. pneumoniae* phages showed a narrow host range [54,55]. The effect of the KPP-5 phage depends on the presence of specific b receptors located in the bacterial cell and other factors that determine the ability of the bacteriophages to multiply within their hosts [56]. An explanation of why the KPP-5 phage was able to infect all *K. pneumoniae* strains selectively in terms of the host range is possibly due to infecting the capsular type of *K. pneumoniae* [57]. This reinforces the suggestion that capsule depolymerase enzymes are contained in the KPP-5 phage, which enables the identification and digestion of particular capsular types. Previous studies have found that most *K. pneumoniae* phages encode depolymerases that can digest the *Klebsiella* capsule [58]. Polysaccharide depolymerase degrades the macromolecular carbohydrate that industrializes the capsule enclosing the bacterial cell wall [54], followed by peptidoglycan hydrolases, which break down the layer of peptidoglycan to penetrate the cell wall and enter the cytoplasm to enable the phage to transfer its genetic material [54].

The TEM image showed that the KPP-5 phage had an icosahedral head (36.25 nm in diameter) with a short tail (12.59 nm in length), so that the KPP-5 phage had a podovirus morphology (Figure 1B). In line with our findings, a recent study by Tan et al. [59] showed that two phages (phage 117 and phage 31) with short tails and icosahedral heads were isolated against all MDR *K. pneumoniae*.

### 3.2. One-Step Growth Curve and Thermal and pH Stability of KPP-5 Phage

**Figure 2:**
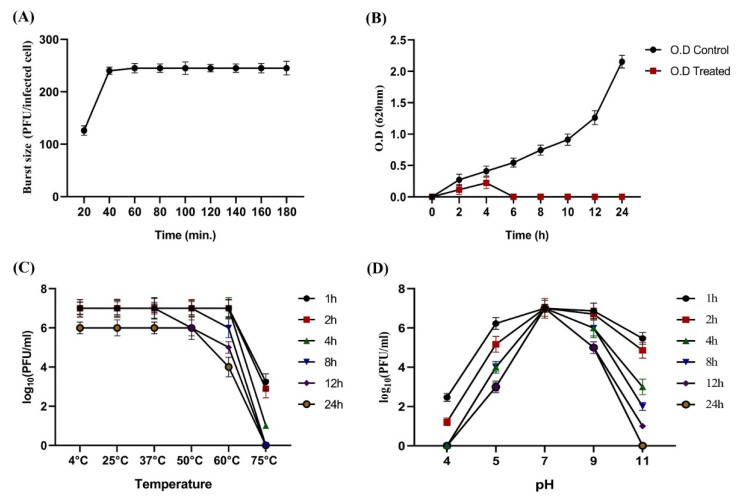
Single-step growth curve of the KPP-5 phage; values of the burst size and latency period were represented vertically and horizontally, respectively (**A**). Titers stability of the KPP-5 phage at different time periods (**B**) under different temperatures (**C**) and under different pH values (**D**).

The one-step growth curve exhibited that the KPP-5 phage had a relatively short latent period of 25 min, and the burst size was about 236 PFU/infected cells (Figure 2A). The short latent period and the high burst size are most likely due to the high processivity of phage DNA polymerase and, therefore, the high replication efficiency [60,61]. According to our findings, the latency period of the KPP-5 phage (25 min) was longer than that of the *Klebsiella* phage ϕBO1E (*Podoviridae*; 10 min) [53]. Fortunately, the burst size of KPP-5 (236 phage particles/infected cells) was higher than that of other *Klebsiella* phages from the *Siphoviridae* [62] and *Podoviridae* [53] families. In comparison to a previous study by Tabassum et al. [63], the latent period for the TSK1 phage was 30 min, with a moderate burst size of 113 PFU/infected cells, indicating that the KPP-5 phage is more effective. Our results indicate that the replication properties of the KPP-5 phage are similar to that of phage B5055 [64]. The bacterial growth reduction tendency of the KPP-5 phage was measured over 24 h of incubation, and the log growth reduction was from the start to six hours of incubation. Then, the bacterial growth remained lower than the undetectable range over the remainder of the experiment time (Figure 2B). This result clearly indicated that there was no development of bacteriophage-resistant mutants due to lack of resistance in bacteria against the phage. Our explanation for the lack of resistance to bacteria may be that the bacteria have not behaved violently, like the formation of proteins that mask the recognition of phage receptor sites, the digestion of the phage genome, or inhibit the action of phage enzymes. It may have other interpretations that require further investigation [65].

On the other hand, the KPP-5 phage showed high titer stability at different temperatures and pH levels, where the phage titer showed high stability after incubation at 4, 25, 37, and 50 °C for all periods of time while phage titer reduction (2.38 log_10_ PFU/mL) was observed at 60 °C after incubation for 24 h. However, more than half of the phage titer (3.76 log_10_ PFU/mL) was reduced for one hour of incubation at 75 °C, and no stable titer was observed at 8, 12, and 24 h of incubation (Figure 2C). Since temperature plays a key role in the bacteriophage survival capacity of the attachment and the length of the latent period [66], a previous study in line with our results showed that the *Caudovirales* bacteriophages were thermostable [67]. Similarly, the acidity and alkalinity of the environment are other important factors affecting the phage stability. The KPP-5 phage showed the highest titer stability at pH 7, while a phage titer reduction was detected at pH 5 and pH 9. Likewise, at pH 11, the phage titer showed a severe reduction of approximately 4.45 and 5.68 (log_10_ PFU/mL) after 8 and 12 h of incubation, while the complete loss of a phage titer was observed at pH 4 and pH 11 after incubation periods of 4 and 24 h, respectively (Figure 2D). Our study was consistent with Jamalludeen et al. [36], who reported that most phages are able to survive well over a wide pH range (5–9) under physiological conditions that maintain a normal virion structure and stability. Extreme pH conditions affect bacteriophage activity by irreversible precipitation, coagulation, or shaking of the phages [68].

### 3.3. Genomic Analysis and Annotation of the KPP-5 Phage

**Figure 3:**
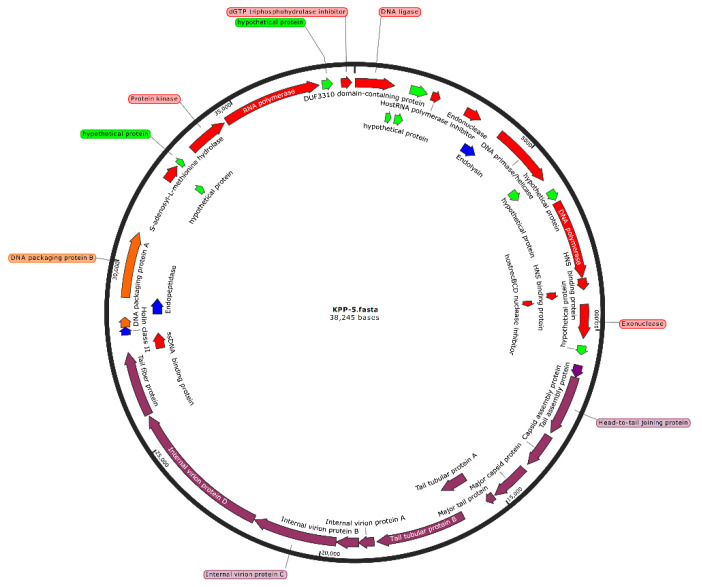
Genome map of *Klebsiella* phage KPP-5. The open reading frames (ORFs) are indicated by specific colors according to their functional categories. Red: replication, regulation, transcription, and translation-related genes. Blue: host cell lysis-related genes. Purple: phage structure-related genes. Orange: DNA packaging-related genes. Green: hypothetical genes.

In general, a bioinformatics analysis can predict both the biological properties and the safety of phage to be used for medical purposes. As a result, the complete KPP-5 phage genome was sequenced and analyzed using various bioinformatics tools. The KPP-5 phage has a linear dsDNA genome of 38,245 bp in length with a GC content of 50.8%. The genome contains 40 predicted open reading frames (ORFs), with the primary starting codon for all ORFs being ATG, all of which have been shown to be located on the KPP-5 phage genome′s positive strand (Figure 3). Of the 40 ORFs predicted, only 31 are identical to the actual functional genes that have been shown to encode proteins, while the remaining nine ORFs were predicted to encode hypothetical proteins with no assigned function. No tRNA genes have been identified in the KPP-5 phage genome. The characteristics of the KPP-5 phage gene products, including ORF positions, predicted products (aa), and homologs with predicted protein motifs, are shown in Table S2. The total annotated ORFs can be further categorized into five modules (Table S3): (i) replication, regulation, transcription, and translation (14 ORFs); (ii) host cell lysis (3 ORFs); (iii) phage structure (12 ORFs); (iv) DNA packaging (2 ORFs); and (v) unknown functions (9 ORFs). Interestingly, phage-borne virulence genes or drug resistance genes were not present, suggesting that KPP-5 could be safely used as a phage biocontrol agent where phage genomes used for therapy should be absolutely safe [69].

### 3.4. Comparative Genomic Analysis

**Figure 4:**
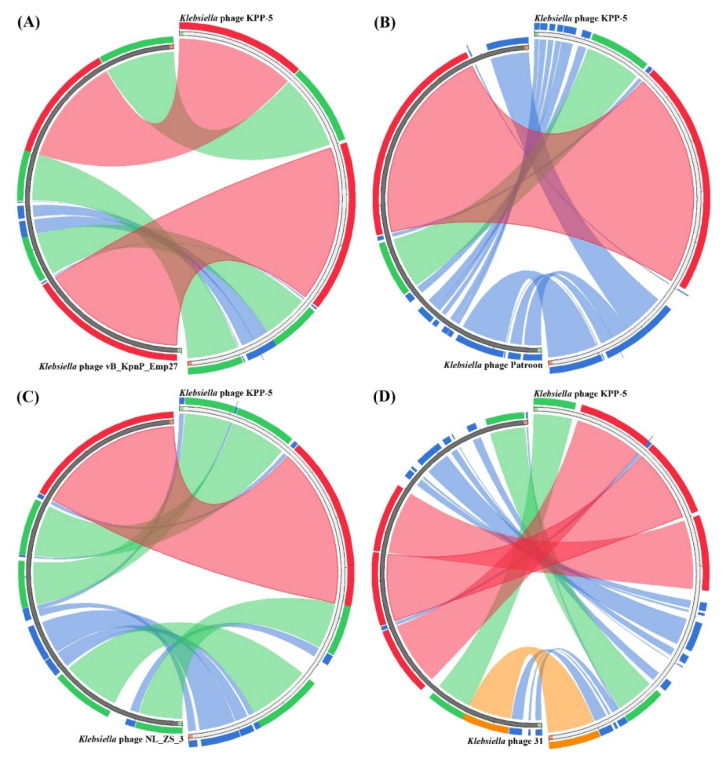
Circos plot depicting sequence similarities of *Klebsiella* phage Kpp-5 against *Klebsiella* phage vB_KpnP_Emp27 (**A**), *Klebsiella* phage Patroon (**B**), *Klebsiella* phage NL_ZS_3 (**C**), and *Klebsiella* phage 31 (**D**). The red color signifies a high sequence similarity followed by orange, green, and blue. Ratio coloring with blue ≤ 0.25, green ≤ 0.50, orange ≤ 0.75, and red > 0.75.

**Figure 5:**
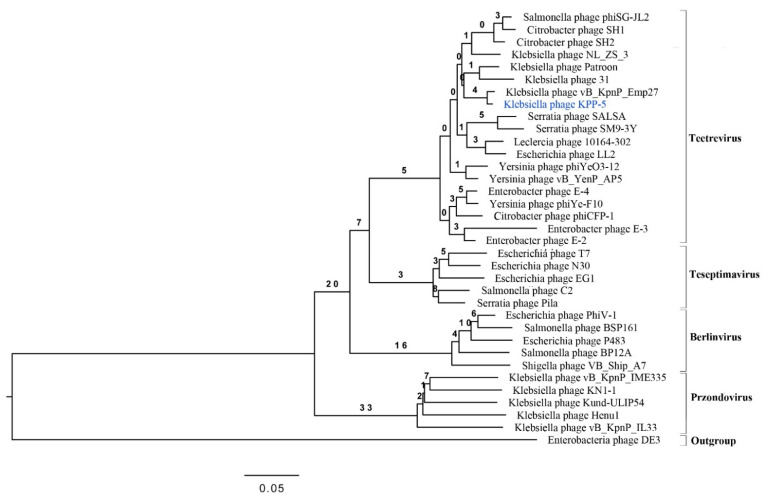
Phylogenetic relations of *Klebsiella* phage KPP-5 based on the whole genome sequence generated by VICTOR.

For a comparative genomic analysis, the genome of the KPP-5 phage was searched against the phage genome sequences in the GenBank using BLASTN in NCBI, where 33 phages belonging to the *Autographiviridae* family and four genera (*Teetrevirus* (*n* = 18), *Teseptimavirus* (*n* = 5), *Berlinvirus* (*n* = 5), and *Przondovirus* (*n* = 5)) were used for analysis (Table S4). The comparative genomic analysis identified KPP-5 phage as belonging to the *Teetrevirus* genus with a high nucleotide sequence similar to the phages in this genus (identity, 85.74%-96.41% and query coverage, 79–99% with E value 0) (Table S4). Of these 18 phages, *Klebsiella* phage vB_KpnP_Emp27 (GenBank accession no. MN013074), *Klebsiella* phage Patroon (GenBank accession no. MK608335), and *Klebsiella* phage NL_ZS_3 (GenBank accession no. MT813142) are closely related to KPP-5 with 99%, 95%, and 94% query coverage, respectively, and 95.19%, 94.85%, and 96.41% complete genome nucleotide sequence identity, respectively (Figure 4A–C). On the other hand, *Klebsiella* phage 31 (GenBank accession no. MN149904) was similar to KPP-5 with 93% query coverage and 85.74% complete genome nucleotide sequence identity (Figure 4D). The average nucleotide identity (ANI) values were calculated using the reciprocal best hits (two-way ANI) between two genomic datasets, as calculated by Goris et al. [70] to further evaluate the relationship between KPP-5 and phage vB_KpnP_Emp27, phage Patroon, phage NL_ZS_3, and phage 31. Since the ANI values were 94.85%, 95.20%, 95.89%, and 95.52%, respectively, this shows that KPP-5 phage is a member of the new species. A phylogenetic analysis of the whole KPP-5 genome using the VICTOR web service revealed five clusters of four genera (*Teetrevirus*, *Teseptimavirus*, *Berlinvirus*, and *Przondovirus*) within the *Autographviridae* family and outgroup within the *Siphoviridae* family (Enterobacteria phage DE3). KPP-5 phage is most closely associated with the *Teetrevirus* genus in the *Autographviridae* family, where it was found to be the closest relative to *Klebsiella* phage vB_KpnP_Emp27 (GenBank accession no. MN013074) (Figure 5). It is therefore suggested that the KPP-5 phage be classified into the *Autographviridae* family and the *Teetrevirus* genus.

The core genes of KPP-5, vB_KpnP_Emp27, Patroon, NL_ZS_3, and 31 phages were examined, and 34 genes were conserved in their genomes (Table S5), while 36 genes were found to be conserved in the genomes of four high-identity phages, including phage KPP-5, vB_KpnP_Emp27, Patroon, and NL_ZS_3 (Table S5). Interestingly, 37 genes were found to be conserved in the genomes of the two KPP-5 and vB_KpnP_Emp27 phages that exhibited high identity and query coverage (Table S). The gene products of these 37 genes were 14 predicted to encode proteins for replication, regulation, transcription, and translation; 3 predicted to encode proteins for host cell lysis; 12 predicted to encode proteins for the phage structure; 2 predicted to encode proteins for DNA packaging; and 6 predicted to encode proteins for unknown functions (hypothetical proteins) (Tables S3 and S5). Three distinct genes in the KPP-5 phage genome were shown in the comparison between the KPP-5 and vB-KpnP-Emp27 phage genomes, including *gp*2 (GenBank No. QSJ04722), *gp*9 (GenBank No. QSJ04729), and *gp*35 (GenBank No. QSJ04755), which were predicted to encode hypothetical proteins (Tables S3 and S5).

### 3.5. Specific Features of the KPP-5 Phage Genome

#### 3.5.1. Replication, Regulation, Transcription, and Translation-Related Genes

The genomic annotation of the phage revealed that 14 of the phage genes were associated with replication, regulation, transcription, and translation, including DNA ligase (*gp*1, GenBank No. QSJ04721), host RNA polymerase inhibitor (*gp*5, GenBank No. QSJ04725), endonuclease (*gp*6, GenBank No. QSJ04726), DNA primase/helicase (*gp*8, GenBank No. QSJ04728), DNA polymerase (*gp*11, GenBank No. QSJ04731), HNS-binding proteins *gp*12 and *gp*13, GenBank Nos. QSJ04732 and QSJ04733, respectively), host recBCD nuclease inhibitor (*gp*14, GenBank No. QSJ04734), exonuclease (*gp*15, GenBank No. QSJ04735), ssDNA-binding protein (*gp*29, GenBank No. QSJ04749), S-adenosyl-L-methionine hydrolase (*gp*34, GenBank No. QSJ04754), protein kinase (*gp*37, GenBank No. QSJ04757), RNA polymerase (*gp*38, GenBank No. QSJ04758), and dGTP triphosphohydrolase inhibitor (*gp*40, GenBank No. QSJ04760) (Table S3).

**Figure 6:**
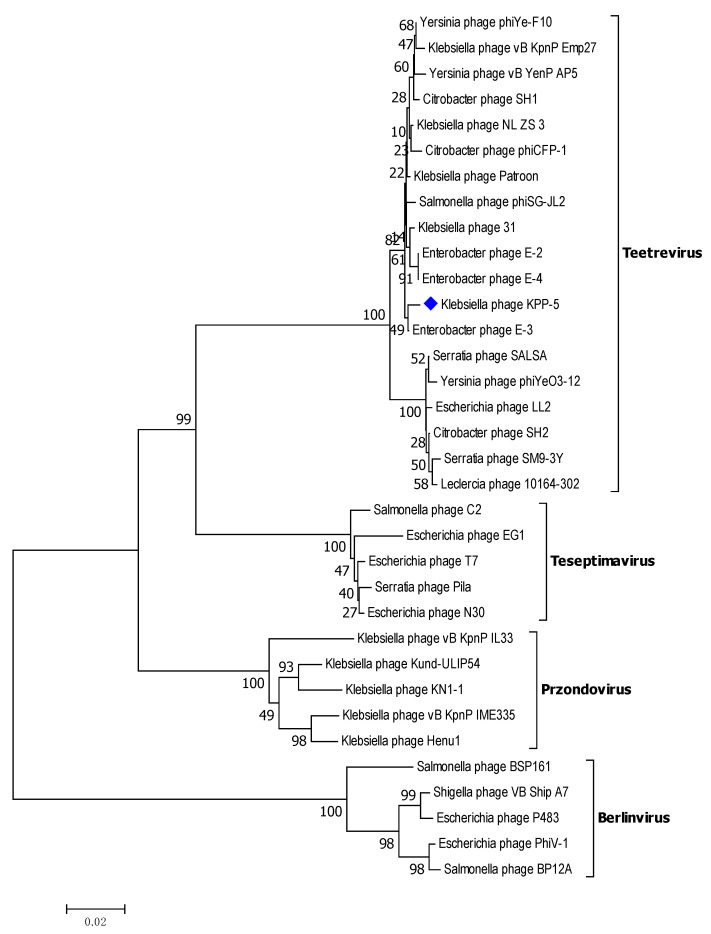
Neighbor-joining tree of *Klebsiella* phage KPP-5 compared to other phages available in GenBank based on the alignment of DNA polymerase amino acid sequences. The numbers represent bootstrap percentage values based on 1000 replicates.

DNA polymerase is a conserved protein frequently used in the analysis of the diversity and global spread of podophages [61]. Therefore, to observe their relationships in greater detail, we compared KPP-5 phage DNA polymerase with the same phages used in the entire genome phylogeny (Table S4). A DNA polymerase phylogenetic analysis showed that KPP-5 is most closely related to the *Teetrevirus* genus in the *Autographviridae* family (Figure 6). The DNA polymerase (*gp*11, GenBank No. QSJ04731) showed a high similarity to the DNA polymerase of the *Enterobacter* phage E-3 (99.57%, GenBank No. YP_009198319), *Klebsiella* phage Patroon (99.29%, GenBank No. QBQ72890), *Klebsiella* phage NL_ZS_3 (99.15%, GenBank No. QNN97360), *Klebsiella* phage 31 (99.15%, GenBank No. QGH73735), and *Klebsiella* phage vB_KpnP_Emp27 (98.72%, GenBank No. QEG11880). Table S5 shows the similarity of 14 genes of *Klebsiella* phage KPP-5 with genes similar to those of *Klebsiella* phage vB KpnP Emp27, *Klebsiella* phage Patroon, *Klebsiella* phage NL-ZS-3, and *Klebsiella* phage 31.

#### 3.5.2. Host Cell Lysis Related Genes

**Figure 7:**
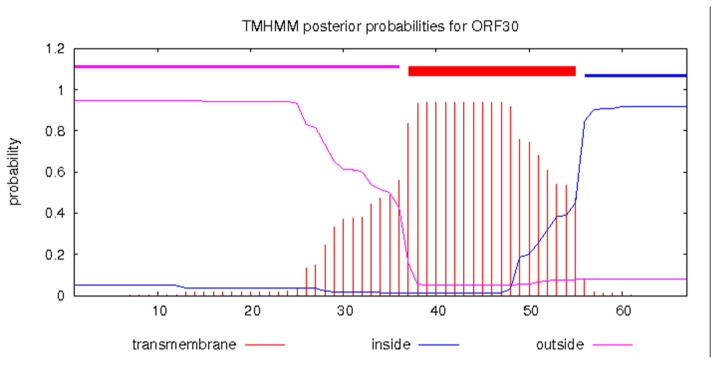
Predicted holin class II (*gp*30, GenBank No. QSJ04750) transmembrane structure using the TMHMM prediction server. Red blocks are predicted transmembrane domains on the top line, while the abscissa represents the sequence position, and the ordinate represents the prediction probability.

Three genes were identified as involving enzymatic cleavage, one encoding endolysin (*gp*7, GenBank No. QSJ04727), the second encoding holin (*gp*30, GenBank No. QSJ04750), and the third encoding endopeptidase (*gp*32, GenBank No. QSJ04752), typically a three-component lysis system for host cell phage release out of the 40 genes predicted in the KPP-5 phage genome. KPP-5 phage endolysin showed a high similarity to *Klebsiella* phage 31 (100%, GenBank No. QGH73730) compared to 99.34%, 96.69%, and 95.36% for *Klebsiella* phage vB_KpnP_Emp27 (GenBank No. QEG11876), *Klebsiella* phage Patroon (GenBank No. QBQ72885), and *Klebsiella* phage NL_ZS_3 (GenBank No. QNN97354), respectively, whereas KPP-5 phage holin showed 100% similarity to *Klebsiella* phage vB_KpnP_Emp27 (GenBank No. QEG11856), *Klebsiella* phage NL_ZS_3 (GenBank No. QNN97335), and *Klebsiella* phage 31 (GenBank No. QGH73755) compared to 89.55% for *Klebsiella* phage Patroon (GenBank No. QBQ72911) (Tables S2 and S5). On the other hand, KPP-5 phage endopeptidase showed a high similarity with sequence identity (154/154) to *Citrobacter* phage SH1 (100%, GenBank No. YP_009286673), followed by sequence identity (150/50) to *Klebsiella* phage NL_ZS_3 (100%, GenBank No. QNN97337) and sequence identity (132/132) to *Klebsiella* phage vB_KpnP_Emp27 (100%, GenBank No. QEG11858) (Tables S2 and S5). Endolysins are distinct peptidoglycan hydrolases and, in various infectious models, have tremendous potential as effective enzybiotics [71]. Four classes are classified as endolysins: muramidase, endopeptidases, amidase, and lytic transglycosylases [71,72]. The endolysin of phage KPP-5 was found to be 16.88 KDa (Table S2), where most Gram-negative endolysin-infecting bacteria typically represent globular proteins of 15–20-kDa single domains [71,73]. A T7 phage endolysin (N-acetylmuramoyl-L-alanine amidase) is a 17-kDa protein that, by hydrolyzing the amide bond between the L-alanine and N-acetylmuramoyl residues of the peptidoglycan layer, lyses a variety of Gram-negative [71]. Holins regulate the access of phage-encoded endolysins to peptidoglycan by accumulating and developing lesions in the cytoplasmic membrane, thus triggering host cell lysis at a specific time point [71,72,74]. It was noted that KPP-5 phage holin was classified as class II and contained a single hydrophobic transmembrane domain (TMD) region via the prediction of the TMHMM server (Figure 7). Depending on their topology, holins are divided into three categories (classes I-III), in which all holins have at least one TMD [75].

#### 3.5.3. Phage Structure Related Genes

Six protein-encoding genes associated with the structural tail, including tail assembly protein (*gp*17, GenBank No. QSJ04739), head-to-tail joining protein (*gp*18, GenBank No. QSJ04737), major tail protein (*gp*21, GenBank No. QSJ04741), tail tubular protein A (*gp*22, GenBank No. QSJ04742), tail tubular protein B (*gp*23, GenBank No. QSJ04743), and tail fiber protein (*gp*28, GenBank No. QSJ04748), were annotated among the 12 actual phage structure genes of phage KPP-5 (Table S3). Most likely, they are involved in tail assembly or phage penetration during infection through the host cell′s external membrane [76]. KPP-5 phage tail fiber protein showed high similarity to *Klebsiella* phage vB_KpnP_Emp27 (94.38% identity, and 98% query coverage, GenBank No. QEG11854) compared to this in *Klebsiella* phage NL_ZS_3 (70.49% identity, and 51% query coverage, GenBank No. QNN97333), *Klebsiella* phage Patroon (51.89% identity, and 89% query coverage, GenBank No. QBQ72909), and *Klebsiella* phage 31 (51.89% identity, and 89% query coverage, GenBank No. QGH73753) (Table S5). Interestingly, there was a match (E-value 9.24e-140) to the superfamily of phage T7-like tail fiber protein (PHA00430) and to the superfamily of phage T7 tail fiber protein with E-value 2.36e-63 (pfam03906) [77,78]. Further analysis of KPP-5 phage tail fiber protein using the Phyre2 server [79] revealed that it exhibited structural similarity to the phage T7 tail fiber protein (confidence, 99.1% and identity, 34%) [80]. The tail fiber protein can accurately recognize and bind to the host surface receptors and can mutate throughout evolution, contributing to a shift in the phage host′s range [81]. Interestingly, KPP-5 phage tail tubular protein A (TTPA) showed a high similarity to *Yersinia* phage phiYeO3-12 (100% identity and 100% query coverage, GenBank No. NP_052110). In view of the hydrolytic activity of the *Yersinia* phage phiYeO3-12 TTPA towards Red starch, it has been shown that this enzyme could be classified within the alpha-1, 4-glucosidase family [82]. Further analysis of KPP-5 phage TTPA using the Phyre2 server [79] revealed that it was structurally similar to the T7 gatekeeper protein (gp11) structure (confidence, 100% and identity, 80%) [83] and to *klebsiella pneumoniae* phage kp32 TTPA, which has lytic activity towards capsular exopolysaccharide (EPS) (confidence, 100% and identity, 61%) [84], since the use of phages is promising for the destruction of bacterial biofilm via depolymerases [84,85,86]. Depolymerases may be found in the tail of the phage or may exist as an extracellular enzyme [84]. *Klebsiella pneumoniae* phage kp32 TTPA exhibited lytic activity towards capsular exopolysaccharide of the multi-resistant clinical strain of *Klebsiella pneumoniae*, PCM2713 where the enzymatic activity of TTPA may reflect the presence of a peptidoglycan hydrolase domain in the α-helical region [84]. Furthermore, protein folding and aggregation tests have shown that *Yersinia* phage phiYeO3-12 TTPAgp11 is a single-domain protein that may be aggregated by maltose or N-acetylglucosamine [82].

**Figure 8:**
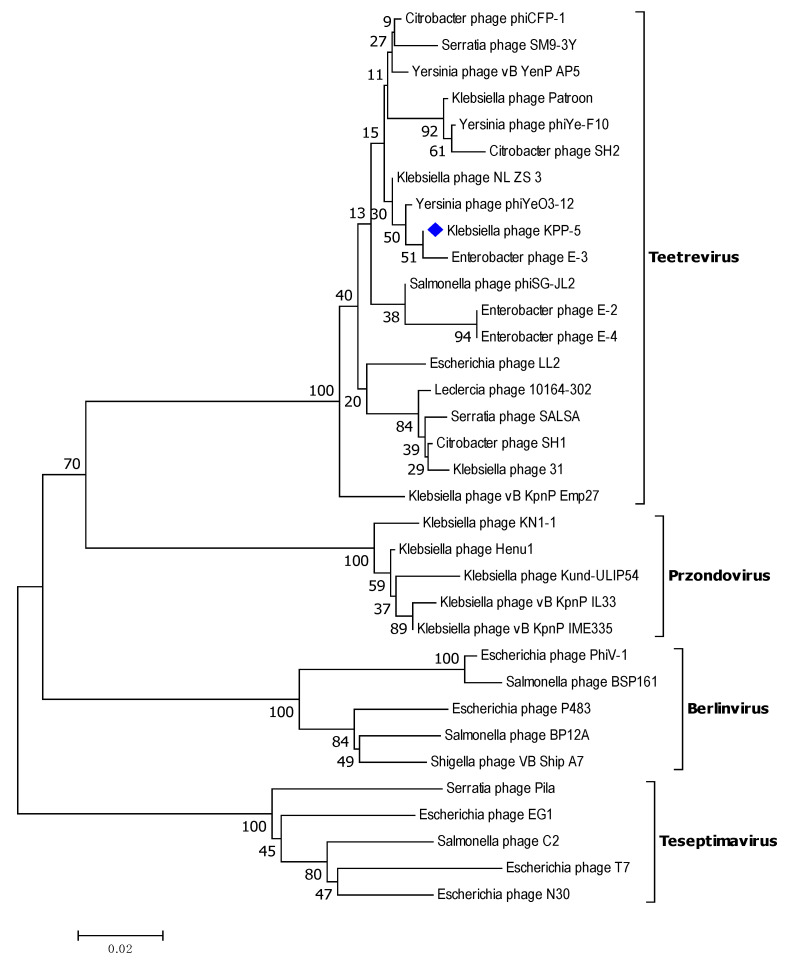
Neighbor-joining tree of *Klebsiella* phage KPP-5 compared to other phages available in GenBank based on the alignment of major capsid protein amino acid sequences. The numbers represent bootstrap percentage values based on 1000 replicates.

Six structural protein-encoding genes have also been annotated, including capsid assembly protein (*gp*19, GenBank No. QSJ04738), major capsid protein (*gp*20, GenBank No. QSJ04740), internal virion protein A (*gp*24, GenBank No. QSJ04744), internal virion protein B (*gp*25, GenBank No. QSJ04745), internal virion protein C (*gp*26, GenBank No. QSJ04746), and internal virion protein D (*gp*27, GenBank No. QSJ04747), among the 12 actual phage structure genes of phage KPP-5 (Table S3). KPP-5 phage, a major capsid protein, showed high similarity to *Klebsiella* phage NL_ZS_3 (99.71% identity and 100% query coverage, GenBank No. QNN97371) compared to this in *Klebsiella* phage Patroon (97.99% identity and 100% query coverage, GenBank No. QBQ72901), *Klebsiella* phage vB_KpnP_Emp27 (97.38% identity and 99% query coverage, GenBank No. QEG11891), and *Klebsiella* phage 31 (97.38% identity and 98% query coverage, GenBank No. QGH73746) (Table S5). The major capsid protein is relatively conserved and is often used to classify phages [87,88,89,90]. Therefore, on the basis of the alignment of major capsid protein amino acid sequences, there were phylogenetic relationship between *Klebsiella* phage KPP-5 and the same phages used in the entire genome phylogeny (Table S4). A major capsid protein phylogenetic analysis showed that KPP-5 is most closely related to the *Teetrevirus* genus in the *Autographviridae* family (Figure 8).

#### 3.5.4. DNA Packaging-Related Genes

**Figure 9:**
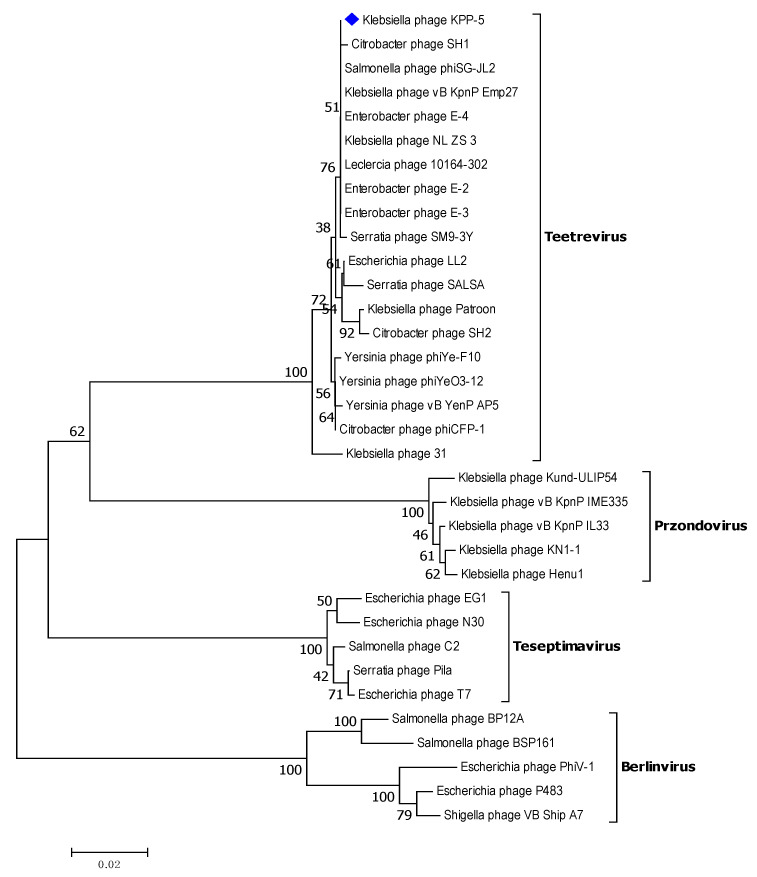
Neighbor-joining tree of *Klebsiella* phage KPP-5 compared to other phages available in GenBank based on the alignment of terminase large subunit amino acid sequences. The numbers represent bootstrap percentage values based on 1000 replicates.

Two genes, including DNA packaging protein A (terminase small subunit, *gp*31, GenBank No. QSJ04751) and DNA packaging protein B (terminase large subunit, *gp*33, GenBank No. QSJ04753), were annotated as phage genome packaging-associated genes in the KPP-5 phage genome (Table S3). During the phage replication process, the dsDNA needs to be packaged, and this requires the ATP-guided terminase proteins responsible for slicing dsDNA into the final genome size sequences, followed by placing them into an empty capsid [91,92]. The phage KPP-5 terminase small subunit is highly similar to the terminase small subunit of *Klebsiella* phage Patroon and *Klebsiella* phage 31 (100% identity and 100% query coverage) (Table S5), while the phage KPP-5 terminase large subunit is highly similar to the terminase large subunit of *Klebsiella* phage vB_KpnP_Emp27 and *Klebsiella* phage NL_ZS_3 (100% identity, 100% query coverage) (Table S5). The terminase large subunit is often used to classify phages, because it is generally well-conserved in tailed phages where phages with a similar gene would assume that their terminase large subunit would be clustered together [93]. Therefore, on the basis of the alignment of the terminase large subunit amino acid sequences, the phylogenetic relationship between *Klebsiella* phage KPP-5 and the same phages used in the entire genome phylogeny (Table S4), the terminase large subunit phylogenetic analysis showed that KPP-5 is most closely related to the *Teetrevirus* genus in the *Autographviridae* family (Figure 9).

#### 3.5.5. Related Unknown Function Genes

**Figure 10:**
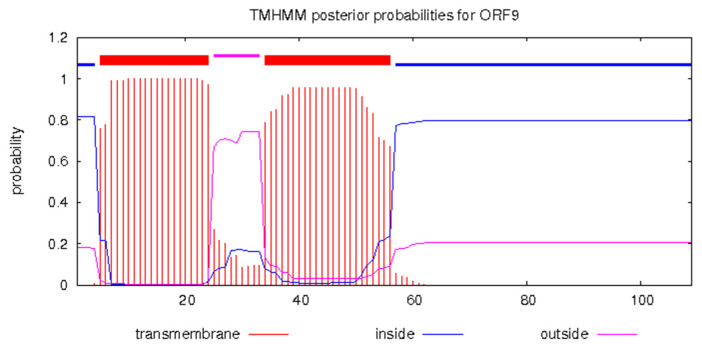
Predicted hypothetical protein (*gp*09, GenBank No. QSJ04729) transmembrane structure using the TMHMM prediction server. Red blocks are predicted transmembrane domains on the top line, while the abscissa represents the sequence position, and the ordinate represents the prediction probability.

It was predicted that nine genes (*gp*2, *gp*3, *gp*4, *gp*9, *gp*10, *gp*16, *gp*35, *gp*36, and *gp*39 with GenBank Nos. QSJ04722, QSJ04723, QSJ04724, QSJ04729, QSJ04730, QSJ04736, QSJ04755, QSJ04756, and QSJ04759, respectively) would encode hypothetical unassigned function proteins among the 40 *Klebsiella* phage KPP-5 genes (Table S3). Of the nine genes predicted, only seven were homologous to other phage-encoded uncharacterized proteins, and two (*gp*2 and *gp*35) were detected only in the KPP-5 phage genome (Table S2). Furthermore, six out of seven genes were homologous to the hypothetical proteins of *Klebsiella* phages, where five genes (*gp*3, *gp*4, *gp*10, *gp*16, and *gp*39) were homologous to *Klebsiella* phage vB_KpnP_Emp27, *Klebsiella* phage Patroon, *Klebsiella* phage NL_ZS_3, and *Klebsiella* phage 31, while *gp*36 was homologous to *Klebsiella* phage vB_KpnP_Emp27, *Klebsiella* phage Patroon, and *Klebsiella* phage NL_ZS_3 (Table S5). Interestingly, *gp*9 was highly similar to the hypothetical protein of *Citrobacter* phage SH1 (97.25% identity and 100% query coverage, GenBank No. YP_009286649) contained double TMD regions via the prediction of the TMHMM server (Figure 10). In phage genomes, 70% of the predicted genes are annotated to encode hypothetical proteins, most of which range from 30 to 200 amino acids with unknown functions [18,94,95,96]. The high potential for the use of these proteins as toxic proteins has already been demonstrated, where they can be toxic through unknown mechanisms and have the potential to develop into novel antimicrobial peptides [18].

## 4. Conclusions

In conclusion, we identified a new phage named KPP-5 capable of lysing MDR *K. pneumoniae* isolated from food samples in Egypt. Based on our findings, the KPP-5 phage is a promising candidate for the control of *Klebsiella* strains and could be safely used as a biocontrol agent for phage-borne virulence genes or for drug resistance genes in their genomes.

## Figures and Tables

**Table 1:** KPP-5 phage host range spectrum and EOP activity.

Strains	Source	KPP-5 Phage Isolate	
		ST	EOP
*K. pneumoniae*-1 (host strain)	food	+	H
*K. pneumoniae*-2	food	+	M
*K. pneumoniae*-3	food	+	M
*K. pneumoniae*-4	food	+	H
*K. pneumoniae*-5	food	+	H
*K. pneumoniae*-6	clinical	+	H
*K. pneumoniae*-7	clinical	+	H
*K. pneumoniae*-8	clinical	+	M
*K. pneumoniae*-9	clinical	+	H
*K. pneumoniae*-10	clinical	+	M
*K. pneumoniae*-11	clinical	+	H
*K. pneumoniae*-12	clinical	+	H
*K. pneumoniae*-13	clinical	+	H
*K. pneumoniae*-14	clinical	+	H
*K. pneumoniae*-15	clinical	+	H
*K. pneumoniae*-16	clinical	+	H
*K. pneumoniae*-17	clinical	+	M
*K. pneumoniae*-18	clinical	+	H
*K. pneumoniae*-19	clinical	+	H
*E. coli*-1	clinical	−	N
*E. coli*-2	clinical	−	N
*E. coli*-3	clinical	−	N
*E. coli*-4	clinical	−	N
*E. coli*-5	clinical	−	N
*E. coli*-6	clinical	−	N
*S.* Typhimurium-1	food	−	N
*S.* Typhimurium-2	food	−	N
*S.* Typhimurium-3	food	−	N
*P. aeruginosa*-1	clinical	−	N
*P. aeruginosa*-2	clinical	−	N
*P. aeruginosa*-3	food	−	N

ST: spotting test, +: positive result (phage infects the bacterial strain), −: negative result (phage does not infect the bacterial strain), EOP; efficiency of plating, H: high EOP from 0.5–1.0, M: moderate EOP from 0.2–0.4, L: low EOP from 0.001–0.1, and N: no EOP (inefficient) <0.001.

## Data Availability

Not applicable.

## Supplementary Materials

The following are available online at https://www.mdpi.com/article/10.3390/biomedicines9040342/s1: Table S1: Antibiotics resistance profiles of the *K. pneumoniae* host strain. Table S2: Genome feature of phage KPP-5, gene products, and their predicted functions. Table S3: KPP-5 phage gene products are involved in five different modules. Table S4: Comparison of phages with BLASTN scores against the KPP-5 phage genome. Table S5: The core genes of *Klebsiella* phage KPP-5, *Klebsiella* phage vB_KpnP_Emp27, *Klebsiella* phage Patroon, *Klebsiella* phage NL_ZS_3, and *Klebsiella* phage 31.
