# Supplementary material for: Characterization and Full Genome Sequence of Novel KPP-5 Lytic Phage against Klebsiella pneumoniae Responsible for Recalcitrant Infection
Source: Biomedicines. 2021 Mar 28;9(4):342. doi: 10.3390/biomedicines9040342 (PMC8066614; doi:10.3390/biomedicines9040342)
Supplement: Supplementary file 1 [file biomedicines-09-00342-s001.pdf]

biomedicines

MDPI

Supplementary

Characterization and Full Genome Sequence of Novel KPP-5 Lytic Phage Against *Klebsiella Pneumoniae* Responsible for Recalcitrant Infection

Ahmed R. Sofy [1,*], Noha K. El-Dougdoug [2], Ehab E. Refaey [1], Rehab A. Dawoud [3,4] and Ahmed A. Hmed [1]

[1] Botany and Microbiology Department, Faculty of Science, Al-Azhar University, Nasr City, Cairo 11884, Egypt; ehabrefaey@azhar.edu.eg (E.E.R.); ahmed_hmed@azhar.edu.eg (A.A.H.)
[2] Botany and Microbiology Department, Faculty of Science, Benha University, Benha 13518, Egypt; nohaeldougdoug@gmail.com
[3] Virus and Phytoplasma Research Department, Plant Pathology Research Institute, Agricultural Research Center (ARC), Giza 12619, Egypt; rdawood@jazanu.edu.sa
[4] Department of Biology, Faculty of Science, Jazan University, Box 114, Jazan 45142, Saudi Arabia
* Correspondence: ahmed_sofy@azhar.edu.eg (A.R.S)

Table S1. Antibiotics resistance profiles of the *K. pneumoniae* host strain.

Scientific Name	Disc Potency (mcg)	Trade Name	Symbol	Antibiotic-resistance profiles
Ampicillin	10	Ampicillin	Am	I
Gentamicin	10	Gentamicin	CN	R
Ciprofloxacin	5	Tarivid	CIP	R
Ofloxacin	5	Tarivid	OFX	I
Oxacillin	1	Oxacillin	OX	S
Clindamycin	2	Augmentin	DA	R
Levofloxacin	5	Lee flox	LEV	I
Flucloxacillin	5	Keflin	FL	R
Rifamycin	30	Remactan	RF	R
Tobramycin	10	Nebcin	TOB	S
Norfloxacin	10	Noroxin	NOR	I
Gatifloxacin	5	Lincocin	GAT	S
Tetracycline	30	Tetracycline	TE	I
Erythromycin	15	Erythromycin	E	R
Aztreonam	1	Meronam	ATM	R
Vancomycin	30	Vancocin	VA	I
Cephradine	30	Velocef	CE	S

Table S2. Genome feature of phage KPP-5, gene products, and their predicted functions.

Gene				Gene product				BLASTp best match	E-value	Identities	Accession
Gene accession	Location	Strand	Frame	Protein size (aa)	Molecule mass (KDa)	Isoelectric Point (pI)	Predicted protein function				
*gp*1 (QSJ04721)	16-1062	+	1	348	39.54	4.99	DNA ligase	*Klebsiella* phage vB_KpnP_Emp27	0	345/348 (99.14%)	QEG11869
*gp*2 (QSJ04722)	950-1126	+	2	58	6.32	12.43	hypothetical protein	-	-	-	-
*gp*3 (QSJ04723)	1232-1489	+	2	85	9.90	11.58	hypothetical protein	*Enterobacter* phage E-3	6e-54	83/85 (97.65%)	YP_009198329
*gp*4 (QSJ04724)	1489-1959	+	1	156	17.67	9.65	DUF3310 domain-containing protein	*Klebsiella* phage vB_KpnP_Emp27	1e-111	154/156 (98.72%)	QEG11871
*gp*5 (QSJ04725)	2080-2316	+	1	78	8.85	4.56	Host RNA polymerase inhibitor, T7-like	*Enterobacter* phage E-3	8e-52	78/78 (100%)	YP_009198326
*gp*6 (QSJ04726)	3066-3527	+	3	153	17.57	10.15	endonuclease	*Enterobacter* phage E-3	3e-107	153/153 (100%)	YP_009198324
*gp*7 (QSJ04727)	3520-3975	+	1	151	16.88	9.23	endolysin	*Klebsiella* phage 31	2e-109	151/151 (100%)	QGH73730
*gp*8 (QSJ04728)	4154-5854	+	2	566	62.80	4.90	DNA primase/helicase	*Enterobacteria* phage T3	0	563/566 (99.47%)	NP_523315
*gp*9 (QSJ04729)	5574-5903	+	3	109	11.93	7.18	hypothetical protein	*Citrobacter* phage SH1	5e-71	106/109 (97.25%)	YP_009286649
*gp*10 (QSJ04730)	6176-6460	+	2	94	10.75	10.60	hypothetical protein	*Klebsiella* phage 31	3e-63	94/94 (100%)	QGH73734
*gp*11 (QSJ04731)	6528-8642	+	3	704	79.76	6.96	DNA polymerase	*Enterobacter* phage E-3	0	701/704 (99.57%)	YP_009198319
*gp*12 (QSJ04732)	8662-8961	+	1	99	11.17	8.48	HNS binding protein	*Enterobacter* phage E-3	3e-66	99/99 (100%)	YP_009198318
*gp*13 (QSJ04733)	8961-9170	+	3	69	7.26	10.25	HNS binding protein	*Klebsiella* phage vB_KpnP_Emp27	2e-42	69/69 (100%)	QEG11882
*gp*14 (QSJ04734)	9167-9346	+	2	59	6.70	3.96	host recBCD nuclease inhibitor	*Enterobacter* phage E-3	3e-35	58/59 (98.31%)	YP_009198316

*gp*15 (QSJ04735)	9343-10254	+	1	303	34.77	4.75	exonuclease	*Klebsiella* phage Patroon	0	302/303 (99.67%)	QBQ72894
*gp*16 (QSJ04736)	10442-10687	+	2	81	9.37	6.24	hypothetical protein	*Yersinia* phage phiYeO3-12	5e-53	81/81 (100%)	NP_052103
*gp*17 (QSJ04739)	10970-11290	+	2	106	11.0	10.58	tail assembly protein	*Klebsiella* phage Patroon	2e-63	105/106 (99.06%)	QBQ72898
*gp*18 (QSJ04737)	11301-12908	+	3	535	58.64	4.27	head-to-tail joining protein	*Yersinia* phage phiYeO3-12	0	535/535 (100%)	NP_052106
*gp*19 (QSJ04738)	13010-13942	+	2	310	33.72	4.02	capsid assembly protein	*Salmonella* phage phiSG-JL2	0	306/310 (99%)	YP_001949781
*gp*20 (QSJ04740)	14099-15142	+	2	347	36.92	6.73	major capsid protein	*Klebsiella* phage NL_ZS_3	0	346/347 (99.71%)	QNN97371
*gp*21 (QSJ04741)	15181-15414	+	1	77	7.44	4.14	major tail protein	*Enterobacter* phage E-3	4e-41	74/77 (96.10%)	YP_009198343
*gp*22 (QSJ04742)	15527-16117	+	2	196	22.23	4.21	tail tubular protein A	*Yersinia* phage phiYeO3-12	1e-142	196/196 (100%)	NP_052110
*gp*23 (QSJ04743)	16133-18538	+	2	801	89.79	6.30	tail tubular protein B	*Citrobacter* phage SH1	0	798/801 (99.50%)	YP_009286665
*gp*24 (QSJ04744)	18611-19021	+	2	136	15.80	5.67	internal virion protein A	*Klebsiella* phage 31	7e-96	134/136 (98.53%)	QGH73749
*gp*25 (QSJ04745)	19024-19617	+	1	197	21.25	9.85	internal virion protein B	Yersinia phage phiYeO3-12	2e-139	197/197 (100%)	NP_052114
*gp*26 (QSJ04746)	19620-21863	+	3	747	85.08	5.32	internal virion protein C	*Klebsiella* phage NL_ZS_3	0	743/747 (99.46%)	QNN97377
*gp*27 (QSJ04747)	21882-25844	+	3	1320	143.52	8.61	internal virion protein D	*Enterobacter* phage E-3	0	1306/1320 (98.94%)	YP_009198337
*gp*28 (QSJ04748)	25892-27622	+	2	576	63.15	5.22	tail fiber protein	*Klebsiella* phage vB_KpnP_Emp27	0	537/569 (94.38%)	QEG11854
*gp*29 (QSJ04749)	27586-28050	+	1	154	17.86	4.51	ssDNA binding protein	*Klebsiella* phage vB_KpnP_Emp27	2e-75	110/137 (80.29%)	QEG11855
*gp*30 (QSJ04750)	28091-28294	+	2	67	7.41	6.72	holin class II	*Klebsiella* phage vB_KpnP_Emp27	3e-40	67/67 (100%)	QEG11856
*gp*31 (QSJ04751)	28298-28564	+	2	88	9.91	4.46	DNA packaging protein A	*Klebsiella* phage Patroon	2e-56	88/88 (100%)	QBQ72912

*gp*32 (QSJ04752)	28642-29106	+	1	154	17.29	9.98	endopeptidase (Rz-like lysis protein)	*Citrobacter* phage SH1	3e-110	154/154 (100%)	YP_009286673
*gp*33 (QSJ04753)	29081-30844	+	2	587	66.72	5.17	DNA packaging protein B	*Klebsiella* phage vB_KpnP_Emp27	0	587/587 (100%)	QEG11859
*gp*34 (QSJ04754)	32419-32877	+	1	152	17.01	7.86	S-adenosyl-L-methionine hydrolase	*Klebsiella* phage Patroon	4e-111	152/152 (100%)	QBQ72866
*gp*35 (QSJ04755)	32687-32884	+	2	65	7.36	10.71	hypothetical protein	-	-	-	-
*gp*36 (QSJ04756)	32941-33099	+	1	52	6.01	9.23	hypothetical protein	*Citrobacter* phage SH2	8e-26	49/52 (94.23%)	YP_009289297
*gp*37 (QSJ04757)	33465-34574	+	3	369	42.53	8.55	protein kinase	*Klebsiella* phage NL_ZS_3	0	325/369 (88.08%)	QNN97343
*gp*38 (QSJ04758)	34645-37299	+	1	884	98.81	7.51	RNA polymerase	*Klebsiella* phage 31	0	881/884 (99.66%)	QGH73719
*gp*39 (QSJ04759)	37386-37658	+	3	90	10.34	9.89	hypothetical protein	*Enterobacter* phage E-2	8e-59	89/90 (98.89%)	YP_009226208
*gp*40 (QSJ04760)	37891-38166	+	1	91	10.48	8.50	dGTP triphosphohydrolase inhibitor	*Klebsiella* phage 31	3e-60	89/91 (97.80%)	QGH73722

Table S3. KPP-5 phage gene products are involved in five different modules.

Modules	Protein	ORFs	Location
**Replication, regulation, transcription, and translation**	DNA ligase	ORF 01	16-1062
	Host RNA polymerase inhibitor	ORF 05	2080-2316
	Endonuclease	ORF 06	3066-3527
	DNA primase/helicase	ORF 08	4154-5854
	DNA polymerase	ORF 11	6528-8642
	HNS binding protein	ORF 12	8662-8961
	HNS binding protein	ORF 13	8961-9170
	host recBCD nuclease inhibitor	ORF 14	9167-9346
	Exonuclease	ORF 15	9343-10254
	ssDNA binding protein	ORF 29	27586-28050
	S-adenosyl-L-methionine hydrolase	ORF 34	32419-32877
	Protein kinase	ORF 37	33465-34574
	RNA polymerase	ORF 38	34645-37299
	dGTP triphosphohydrolase inhibitor	ORF 40	37891-38166
**Host cell lysis**	Endolysin	ORF 07	3520-3975
	Holin class II	ORF 30	28091-28294
	Endopeptidase	ORF 32	28642-29106
**Phage structure**	Tail assembly protein	ORF 17	10970-11290
	Head-to-tail joining protein	ORF 18	11301-12908
	Capsid assembly protein	ORF 19	13010-13942
	Major capsid protein	ORF 20	14099-15142
	Major tail protein	ORF 21	15181-15414
	Tail tubular protein A	ORF 22	15527-16117
	Tail tubular protein B	ORF 23	16133-18538
	Internal virion protein A	ORF 24	18611-19021
	Internal virion protein B	ORF 25	19024-19617
	Internal virion protein C	ORF 26	19620-21863
	Internal virion protein D	ORF 27	21882-25844
	Tail fiber protein	ORF 28	25892-27622
**DNA packaging**	DNA packaging protein A	ORF 31	28298-28564
	DNA packaging protein B	ORF 33	29081-30844
**Unknown functions**	hypothetical protein	ORF 02	950-1126
	hypothetical protein	ORF 03	1232-1489
	DUF3310 domain-containing protein	ORF 04	1489-1959
	hypothetical protein	ORF 09	5574-5903
	hypothetical protein	ORF 10	6176-6460
	hypothetical protein	ORF 16	10442-10687
	hypothetical protein	ORF 35	32687-32884
	hypothetical protein	ORF 36	32941-33099
	hypothetical protein	ORF 39	37386-37658

Table S4. Comparison of phages with BLASTN scores against the KPP-5 phage genome.

Phage name	Host	Phage Family	Genus	Location	Collection date	Genome Length (kb)	Accession No.	Query Coverage	E-value	Genome Identity
Klebsiella phage vB_KpnP_Emp27	*Klebsiella pneumoniae*	*Autographiviridae*	*Teetrevirus*	USA	2017	38,603	MN013074	99%	0	16665/17507 (95.19%)
Klebsiella phage Patroon	*Klebsiella pneumoniae*	*Autographiviridae*	*Teetrevirus*	USA	2016	39,442	MK608335	95%	0	22172/23377 (94.85%)
Yersinia phage phiYe-F10	*Yersinia enterocolitica*	*Autographiviridae*	*Teetrevirus*	China	2012	39,210	NC_047755	94%	0	23744/24777 (95.83%)
Enterobacter phage E-4	*Enterobacter cloacae*	*Autographiviridae*	*Teetrevirus*	Portugal	2014	39,142	KP791807	94%	0	13978/14751 (94.76%)
Klebsiella phage NL_ZS_3	*Klebsiella pneumoniae*	*Autographiviridae*	*Teetrevirus*	-	-	38,741	MT813142	94%	0	12490/12955 (96.41%)
Citrobacter phage SH2	*Citrobacter freundii*	*Autographiviridae*	*Teetrevirus*	-	-	39,158	NC_031092	93%	0	15703/17435 (90.07%)
Escherichia phage LL2	*Escherichia coli*	*Autographiviridae*	*Teetrevirus*	USA	-	39,382	NC_048063	93%	0	10890/11340 (96.03%)
Serratia phage SALSA	*Serratia marcescens*	*Autographiviridae*	*Teetrevirus*	Germany	2018	39,933	MT419366	93%	0	10726/11340 (94.59%)
Yersinia phage vB_YenP_AP5	*Yersinia enterocolitica*	*Autographiviridae*	*Teetrevirus*	Canada	-	38,646	NC_025451	93%	0	8263/8721 (94.75%)
Klebsiella phage 31	*Klebsiella pneumoniae*	*Autographiviridae*	*Teetrevirus*	China	-	39,600	MN149904	93%	0	9419/10986 (85.74%)
Yersinia phage phiYeO3-12	*Yersinia enterocolitica*	*Autographiviridae*	*Teetrevirus*	-	-	39,600	NC_001271	93%	0	7168/7485 (95.76%)

Leclercia phage 10164-302	*Leclercia adecarboxylata*	*Autographiviridae*	*Teetrevirus*	-	-	39,300	NC_047843	92%	0	10895/11338 (96.09%)
Citrobacter phage SH1	*Citrobacter freundii*	*Autographiviridae*	*Teetrevirus*	-	-	39,434	NC_031066	92%	0	10463/10886 (96.11%)
Salmonella phage phiSG-JL2	*Salmonella enterica*	*Autographiviridae*	*Teetrevirus*	South Korea	-	38,815	NC_010807	92%	0	10461/10886 (96.10%)
Serratia phage SM9-3Y	*Serratia marcescens*	*Autographiviridae*	*Teetrevirus*	-	-	39,631	NC_047774	91%	0	10555/10992 (96.02%)
Citrobacter phage phiCFP-1	*Citrobacter freundii*	*Autographiviridae*	*Teetrevirus*	China	2013	38,625	NC_028880	90%	0	12489/12988 (96.16%)
Enterobacter phage E-2	*Enterobacter cloacae*	*Autographiviridae*	*Teetrevirus*	Portugal	2014	36,051	NC_029102	89%	0	13978/14751 (94.76%)
Enterobacter phage E-3	*Enterobacter cloacae*	*Autographiviridae*	*Teetrevirus*	Portugal	2014	31,522	NC_028795	79%	0	14035/14572 (96.31%)
Salmonella phage C2	*Salmonella enterica*	*Autographiviridae*	*Teseptimavirus*	South Korea	2016	38,554	MN026740	79%	0	7543/11026 (68.41%)
Escherichia phage N30	*Escherichia coli*	*Autographiviridae*	*Teseptimavirus*	China	-	39,266	NC_048078	78%	0	5183/7599 (68.21%)
Serratia phage Pila	*Serratia marcescens*	*Autographiviridae*	*Teseptimavirus*	USA	2018	38,678	MN098329	78%	0	6588/9633 (68.39%)
Escherichia phage EG1	*Escherichia coli*	*Autographiviridae*	*Teseptimavirus*	China	2016	39,919	NC_047895	78%	0	5257/7648 (68.74%)
Escherichia phage T7	*Escherichia coli*	*Autographiviridae*	*Teseptimavirus*	-	-	39,938	AY264774	78%	0	5192/7603 (68.29%)
Escherichia phage P483	*Escherichia coli*	*Autographiviridae*	*Berlinvirus*	-	-	40,829	NC_028822	71%	0	2926/4006 (73.04%)

Salmonella phage BSP161	*Salmonella enterica*	*Autographiviridae*	*Berlinvirus*	-	-	39,688	NC_048105	71%	0	3060/4241 (72.15%)
Shigella phage VB_Ship_A7	*Shigella flexneri*	*Autographiviridae*	*Berlinvirus*	China	-	40,058	NC_048180	71%	0	2892/3974 (72.77%)
Escherichia phage PhiV-1	*Escherichia coli*	*Autographiviridae*	*Berlinvirus*	-	2019	39461	MT542512	71%	0	2884/3964 (72.75%)
Salmonella phage BP12A	*Salmonella enterica*	*Autographiviridae*	*Berlinvirus*	Canada	2003	39,696	KM366096	71%		2899/3988 (72.69%)
Klebsiella phage Henu1	*Klebsiella pneumoniae*	*Autographiviridae*	*Przondovirus*	China	2017	40,352	NC_048138	69%	0	4619/6644 (69.52%)
Klebsiella phage KN1-1	*Klebsiella pneumoniae*	*Autographiviridae*	*Przondovirus*	Taiwan	-	40,236	NC_048129	69%	0	4523/6482 (69.78%)
Klebsiella phage vB_KpnP_IME335	*Klebsiella pneumoniae*	*Autographiviridae*	*Przondovirus*	China	-	40,361	MN176574	69%	0	4412/6303 (70.00%)
Klebsiella phage Kund-ULIP54	*Klebsiella pneumoniae*	*Autographiviridae*	*Przondovirus*	France	2015	41,109	MK380016	69%	0	3994/5641 (70.80%)
Klebsiella phage vB_KpnP_IL33	*Klebsiella pneumoniae*	*Autographiviridae*	*Przondovirus*	Poland	2014	41,335	NC_047782	69%	0	3995/5643 (70.80%)
Enterobacteria phage DE3	Escherichia coli	*Siphoviridae*	*Lambdavirus*	-	-	42,925	NC_042057	76.50%	0	2031/2655 (6%)

Table S5. The core genes of *Klebsiella* phage KPP-5, *Klebsiella* phage vB_KpnP_Emp27, *Klebsiella* phage Patroon, *Klebsiella* phage NL_ZS_3, and *Klebsiella* phage 31.

***Klebsiella* phage Kpp-5**		***Klebsiella* phage vB_KpnP_Emp27**		***Klebsiella* phage Patroon**		***Klebsiella* phage NL_ZS_3**		***Klebsiella* phage 31**	
**Gene accession**	**Product**	**Accession Number**	**Product [Identities]**	**Accession Number**	**Product [Identities]**	**Accession Number**	**Product [Identities]**	**Accession Number**	**Product [Identities]**
*gp*1 (QSJ04721)	DNA ligase	QEG11869	putative DNA ligase [345/348(99.14%)]	QBQ72877	ATP-dependent DNA ligase [309/348(88.79%)]	QNN97348	ATP-dependent DNA ligase [324/348(93.10%)]	QGH73723	hypothetical protein [343/348(98.56%)]
*gp*3 (QSJ04723)	hypothetical protein	QEG11870	hypothetical protein [75/85(88.24%)]	QBQ72879	hypothetical protein [82/85(96.47%)]	QNN97349	hypothetical protein [76/85(89.41%)]	QGH73724	hypothetical protein [75/85(88.24%)]
*gp*4 (QSJ04724)	DUF3310 domain-containing protein	QEG11871	DUF3310 domain-containing protein [154/156(98.72%)]	QBQ72880	hypothetical protein [129/163(79.14%)]	QNN97350	nucleotide kinase [154/159(96.86%)]	QGH73725	hypothetical protein [153/156(98.08%)]
*gp*5 (QSJ04725)	Host RNA polymerase inhibitor, T7-like	QEG11873	putative host RNA polymerase inhibitor [77/78(98.72%)]	QBQ72881	inhibitor of host bacterial RNA polymerase [49/50(98%)]	QNN97351	bacterial RNA polymerase inhibitor [77/78(98.72%)]	QGH73727	host RNA polymerase inhibitor [77/78(98.72%)]
*gp*6 (QSJ04726)	endonuclease	QEG11875	putative endonuclease [152/153(99.35%)]	QBQ72884	endonuclease I [111/141(78.72%)]	QNN97353	endonuclease I [152/153(99.35%)]	-	-
*gp*7 (QSJ04727)	endolysin	QEG11876	putative N-acetylmuramoyl-L-alanine amidase [150/151(99.34%)]	QBQ72885	endolysin [146/151(96.69%)]	QNN97354	endolysin [144/151(95.36%)]	QGH73730	lysin [151/151(100%)]
*gp*8 (QSJ04728)	DNA primase/helicase	QEG11877	putative primase/helicase [559/566(98.76%)]	QBQ72887	DNA primase/helicase [540/566(95.41%)]	QNN97357	primase/helicase protein [499/504(99.01%)]	QGH73732	hypothetical protein [498/504(98.81%)]
*gp*10 (QSJ04730)	hypothetical protein	QEG11879	hypothetical protein [92/94(97.87%)]	QBQ72889	hypothetical protein [93/94(98.94%)]	QNN97359	inhibitor of host toxin/antitoxin system [92/94(97.87%)]	QGH73734	hypothetical protein [94/94(100%)]
*gp*11 (QSJ04731)	DNA polymerase	QEG11880	putative DNA polymerase [695/704(98.72%)]	QBQ72890	DNA polymerase [699/704(99.29%)]	QNN97360	DNA-directed DNA polymerase [698/704(99.15%)]	QGH73735	DNA polymerase [698/704(99.15%)]

*gp*12 (QSJ04732)	HNS binding protein	QEG11881	putative HNS binding protein [88/99(88.89%)]	QBQ72891	HNS binding protein [47/101(46.53%)]	QNN97361	suppressor of silencing [44/101(43.56%)]	QGH73736	putative HNS binding protein [98/99(98.99%)]
*gp*13 (QSJ04733)	HNS binding protein	QEG11882	hypothetical protein [69/69(100%)]	QBQ72892	hypothetical protein [68/69(98.55%)]	QNN97362	hypothetical protein [66/69(95.65%)]	QGH73738	putative HNS binding protein [67/69(97.10%)]
*gp*14 (QSJ04734)	host recBCD nuclease inhibitor	QEG11883	hypothetical protein [32/49(65.31%)]	QBQ72893	host recBCD nuclease inhibitor [42/59(71.19%)]	QNN97363	inihibitor of recBCD nuclease [42/59(71.19%)]	QGH73739	inihibitor of recBCD nuclease [42/59(71.19%)]
*gp*15 (QSJ04735)	exonuclease	QEG11884	putative exonuclease [295/303(97.36%)]	QBQ72894	exonuclease [302/303(99.67%)]	QNN97364	exonuclease [297/303(98.02%)]	QGH73740	exonuclease [297/303(98.02%)]
*gp*16 (QSJ04736)	hypothetical protein	QEG11886	hypothetical protein [62/64(96.88%)]	QBQ72896	hypothetical protein [79/81(97.53%)]	QNN97366	hypothetical protein [79/81(97.53%)]	QGH73741	hypothetical protein [79/81(97.53%)]
*gp*17 (QSJ04739)	tail assembly protein	QEG11888	putative tail assembly protein [97/113(85.84%)]	QBQ72898	host specificity protein B [105/106(99.06%)]	QNN97368	virion assembly protein [101/106(95.28%)]	QGH73743	host specificity protein [97/106(91.51%)]
*gp*18 (QSJ04737)	head-to-tail joining protein	QEG11889	putative head-to-tail joining protein [532/535(99.44%)]	QBQ72899	head-to-tail joining protein [534/535(99.81%)]	QNN97369	collar, head-to-tail connector protein [534/535(99.81%)]	QGH73744	hypothetical protein [532/535(99.44%)]
*gp*19 (QSJ04738)	capsid assembly protein	QEG11890	putative capsid assembly protein [293/310(94.52%)]	QBQ72900	capsid assembly protein [299/310(96.45%)]	QNN97370	capsid assembly scaffolding protein [305/310(98.39%)]	QGH73745	hypothetical protein [303/310(97.74%)]
*gp*20 (QSJ04740)	major capsid protein	QEG11891	putative minor capsid protein [335/344(97.38%)]	QBQ72901	major capsid protein [342/349(97.99%)]	QNN97371	major capsid protein [346/347(99.71%)]	QGH73746	capsid and scaffold protein [334/343(97.38%)]
*gp*21 (QSJ04741)	major tail protein	QEG11847	putative minor capsid protein [37/70(52.86%)]	-	-	-	-	-	-
*gp*22 (QSJ04742)	tail tubular protein A	QEG11848	putative tail tubular protein A [193/196(98.47%)]	QBQ72903	tail tubular protein A [193/196(98.47%)]	QNN97373	non-contractile tail tubular protein [195/196(99.49%)]	QGH73747	tail tubular protein A [194/196(98.98%)]

*gp*23 (QSJ04743)	tail tubular protein B	QEG11849	putative tail tubular protein B [781/801(97.50%)]	QBQ72904	tail tubular protein B [793/801(98.63%)]	QNN97374	non-contractile tail tubular protein [794/801(99.13%)]	QGH73748	hypothetical protein [795/801(99.25%)]
*gp*24 (QSJ04744)	internal virion protein A	QEG11850	putative internal virion protein A [133/136(97.06%)]	QBQ72905	internal virion protein A [132/136(97.06%)]	QNN97375	scaffold protein [131/136(96.32%)]	QGH73749	internal (core) protein [134/136(98.53%)]
*gp*25 (QSJ04745)	internal virion protein B	QEG11851	putative internal virion protein B [191/197(96.95%)]	QBQ72906	internal virion protein B [195/197(98.98%)]	QNN97376	DNA ejectosome component [193/197(97.97%)]	QGH73750	protein inside capsid B [196/197(99.49%)]
*gp*26 (QSJ04746)	internal virion protein C	QEG11852	putative internal virion protein C [734/747(98.26%)]	QBQ72907	internal virion protein C [737/747(98.66%)]	QNN97377	DNA ejectosome component [743/747(99.46%)]	QGH73751	hypothetical protein [596/747(79.79%)]
*gp*27 (QSJ04747)	internal virion protein D	QEG11853	putative internal virion protein D [1301/1320(98.56%)]	QBQ72908	internal virion protein D [1297/1320(98.26%)]	QNN97332	DNA ejectosome component [1282/1320(97.12%)]	QGH73752	hypothetical protein [1080/1315(82.13%)]
*gp*28 (QSJ04748)	tail fiber protein	QEG11854	putative tail fiber protein [537/569(94.38%)]	QBQ72909	tail fiber protein [274/528(51.89%)]	QNN97333	non-contractile tail fiber protein [215/305(70.49%)]	QGH73753	tail fiber protein [275/530(51.89%)]
*gp*29 (QSJ04749)	ssDNA binding protein	QEG11855	putative ssDNA binding protein [110/137(80.29%)]	QBQ72910	hypothetical protein [40/107(37.38%)]	QNN97334	hypothetical protein [46/105(43.81%)]	QGH73754	hypothetical protein [41/107(38.32%)]
*gp*30 (QSJ04750)	holin class II	QEG11856	putative class II holin [67/67(100%)]	QBQ72911	holin [60/67(89.55%)]	QNN97335	class II holin [67/67(100%)]	QGH73755	class II holin [67/67(100%)]
*gp*31 (QSJ04751)	DNA packaging protein A	QEG11857	putative DNA packaging protein [87/88(98.86%)]	QBQ72912	DNA packaging protein A [88/88(100%)]	QNN97336	terminase small subunit [87/88(98.86%)]	QGH73756	DNA packaging protein A [88/88(100%)]
*gp*32 (QSJ04752)	endopeptidase (Rz-like lysis protein)	QEG11858	putative endopeptidase [132/132(100%)]	QBQ72913	I-spanin [149/150(99.33%)]	QNN97337	endopeptidase Rz [150/150(100%)]	QGH73757	endopeptidase Rz [86/148(58.11%)]
*gp*33 (QSJ04753)	DNA packaging protein B	QEG11859	putative DNA packaging protein [587/587(100%)]	QBQ72915	DNA packaging protein B [582/587(99.15%)]	QNN97338	terminase large subunit [587/587(100%)]	QGH73759.1	DNA packaging protein [578/587(98.47%)]
*gp*34 (QSJ04754)	S-adenosyl-L-methionine hydrolase	QEG11860	putative adenosyl-L-methionine	QBQ72866	S-adenosyl-L-methionine	QNN97340	S-adenosyl-L-methionine	QGH73714	S-adenosyl-L-methionine

			hydrolase [145/152(95.39%)]		hydrolase [152/152(100%)]		hydrolase [151/152(99.34%)]		hydrolase [139/152(91.45%)]
*gp*36 (QSJ04756)	hypothetical protein	QEG11861	hypothetical protein [47/52(90.38%)]	QBQ72868	hypothetical protein [48/52(92.31%)]	QNN97341	hypothetical protein [45/52(86.54%)]	-	-
*gp*37 (QSJ04757)	protein kinase	QEG11864	putative protein kinase [313/370(84.59%)]	QBQ72871	protein kinase [314/370(84.86%)]	QNN97343	serine/threonine kinase [325/369(88.08%)]	QGH73718	hypothetical protein [297/370(80.27%)]
*gp*38 (QSJ04758)	RNA polymerase	QEG11865	putative RNA polymerase [879/884(99.43%)]	QBQ72872	RNA polymerase [875/884(98.98%)]	QNN97344	DNA-directed RNA polymerase [879/884(99.43%)]	QGH73719	DNA-directed RNA polymerase [881/884(99.66%)]
*gp*39 (QSJ04759)	hypothetical protein	QEG11866	hypothetical protein [87/90(96.67%)]	QBQ72873	hypothetical protein [86/90(95.56%)]	QNN97345	hypothetical protein [28/74(37.84%)]	QGH73720	hypothetical protein [87/90(96.67%)]
*gp*40 (QSJ04760)	dGTP triphosphohydrolase inhibitor	QEG11868	putative dGTP triphosphohydrolas e inhibitor [87/91(95.60%)]	QBQ72875	host dGTP triphosphohydrolas e inhibitor [86/90(95.56%)]	QNN97347	hypothetical protein [89/91(97.80%)]	QGH73722	dGTP triphosphohydrolase inhibitor [89/91(97.80%)]
